# Comparative Proteome and Weighted Gene Co-Expression Network Analyses Uncover the Mechanism of Wheat Grain Protein Accumulation in Response to Nitrogen Fertilizer Application

**DOI:** 10.3390/foods14091481

**Published:** 2025-04-24

**Authors:** Beiming Xu, Yuku Jia, Jianchao Feng, Yang Yang, Geng Ma, Yanfei Zhang, Yingxin Xie, Dongyun Ma

**Affiliations:** 1 National Engineering Research Center for Wheat, College of Agronomy, Henan Agricultural University, Zhengzhou 450046, China; xbm970915@163.com (B.X.); 13633969649@163.com (Y.J.); fjc000@163.com (J.F.); yy927810944@163.com (Y.Y.); nymg5135@163.com (G.M.); yfz15838298834@163.com (Y.Z.); xyx183@126.com (Y.X.); 2 National Wheat Technology Innovation Center, Henan Agricultural University, Zhengzhou 450046, China; 3 State Key Laboratory of High-Efficiency Production of Wheat-Maize Double Cropping, Zhengzhou 450046, China

**Keywords:** wheat grain, differentially expressed proteins, gene co-expression network analysis, grain protein accumulation, nitrogen fertilizer application level

## Abstract

This study uses proteomic technology to identify differentially expressed proteins (DEPs) under varying nitrogen fertilizer levels. Additionally, it utilizes weighted gene co-expression network analysis (WGCNA) based on expression data of DEP-coding genes to explore the mechanism by which nitrogen promotes grain protein accumulation. The results indicate that high-nitrogen treatment leads to an increased grain protein content, wet gluten content, stability time, and energy area. In addition, the β-sheet content of the protein secondary structure increased, while the irregular curl content decreased. A total of 285 DEPs were identified under different nitrogen levels, with 172 upregulated proteins in grains under high-nitrogen treatment including storage proteins (8.14%) and proteins involved in nitrogen metabolism (8.72%), defense/stress (11.04%), regulation (26.16%), and transport (5.23%). This suggests that both storage proteins and certain metabolic proteins contribute to dough network formation. WGCNA revealed a strong correlation between the blue module and grain samples, and Gene Ontology analysis indicated that most genes were enriched in response to abscisic acid (ABA) in the “biological process” category. Furthermore, 18 core genes were identified, with most containing ABA response elements, light response elements, and motifs related to storage protein regulation in their promoter regions. Expression analysis of 10 genes and their predicted transcription factors during the grain-filling stage demonstrated higher expression levels under high-nitrogen conditions. This study provides valuable insights into the promotion of grain protein accumulation and dough quality by nitrogen fertilizer application.

## 1. Introduction

The wheat grain protein content and protein components, traditionally classified as albumin, globulin, gliadin, and glutenin based on their solubility in various solvents, play a crucial role in flour processing. Gluten proteins (glutenin and gliadin) are primarily responsible for the viscoelastic properties of dough and the excellent processing characteristics of diverse wheat-based products [1]. While gliadin and glutenin have been categorized as prolamins [2], other proteins such as α-amylase/trypsin inhibitors, puroindolines, and lipid transfer proteins are considered members of small sulfur-rich prolamins based on their amino acid sequences [3]. Non-gluten proteins (albumins and globulins) have been recognized as relevant to dough and baking product quality, and their role in flour-processing quality cannot be overlooked [4,5,6]. Furthermore, albumins and globulins primarily function as enzymes involved in metabolic activity [7], and the addition of enzymes, such as amylases, xylanases, and glucose oxidase, to flour can enhance bread-making quality [8,9].

Nitrogen fertilizer application plays a critical role in enhancing the grain protein content and flour-processing quality. The high crude protein content of wheat grain and superior bread-making quality are often associated with elevated levels of nitrogen fertilizer application. Increased nitrogen application results in higher quantities of glutenin and gliadin protein components [10,11]. Moreover, nitrogen fertilizer application affects the protein content and compositional profile [12,13]. In comparison to conventional cultivation patterns, reduced nitrogen rates significantly downregulate high-molecular-weight glutenin subunits (HMW-GSs), low-molecular-weight glutenin subunits (LMW-GSs), α/β-gliadins, and γ-gliadins [14]. For high grain yields and quality, farmers often apply excessive nitrogen fertilizers. However, surplus nitrogen that plants cannot utilize may leach into the soil or be emitted into the atmosphere, leading to various ecological and environmental issues, including water pollution and increased greenhouse gas emissions [15].

In response to global environmental changes, increasing food demand, and the pursuit of economic benefits, numerous studies have investigated methods to enhance protein accumulation in grains while reducing nitrogen fertilizer application [16]. Characterizing the regulatory network of efficient nitrogen utilization along with identifying related genes has emerged as a significant research focus. The wheat TaPDI gene, which encodes protein disulfide isomerase, plays a role in the accumulation of storage proteins in the endosperm [17]. The transcription factor TaNAC100 binds to GLU-1 promoters and inhibits the accumulation of HMW-GS and other storage proteins [18]. The transcription factor NAM-B1 has been identified as a regulator of grain protein content and other micronutrients in wheat grain [19]. In addition, several studies have also focused on elucidating the mechanisms of nitrogen-enhanced grain storage protein accumulation through investigations into the transcriptome of grains or assessing the expression levels of genes involved in nitrogen metabolism pathways [20,21,22].

However, previous research has primarily focused on storage protein components and their contents. With the application of proteomics technology, a wide range of protein components has been identified in wheat grains and flour [5,23]; these protein components also play an essential role in processing quality [5,6]. Therefore, by distinguishing grain protein components under different nitrogen levels using proteomics technology, this study analyzed the key factors that promote grain protein synthesis through efficient utilization of nitrogen fertilizer by public database screening, weighted gene co-expression network analysis (WGCNA), and hub gene screening and expression verification. By starting with mature grain proteome profiling and progressing to WGCNA of corresponding coding genes of differential protein components, a more robust relationship between gene expression and protein composition may be established, and the results would provide valuable insights into the mechanism of wheat grain protein accumulation and the efficient utilization of nitrogen fertilizer.

## 2. Materials and Methods

### 2.1. Plant Material and Experimental Design

We used the wheat cultivar ‘Kexing 3302’ (KX 3302), known for its strong gluten properties. The wheat seeds were cultivated at the Saide Breeding Experimental Station in Zhengzhou, Henan Province, China. Planting occurred on 18 October 2021, with a density of 225 seeds/m^2^. Each plot comprised 6 rows, measuring 9 m in length and 1.5 m in width. Two nitrogen fertilizer application treatments were implemented: low nitrogen (LN, 0 kg/hm^2^) and high nitrogen (HN, 240 kg/hm^2^). The experiment comprised three replicates for each treatment group, with all treatments receiving K_2_O 135 kg/hm^2^ and P_2_O_5_ 135 kg/hm^2^ before sowing. The nitrogen fertilizer application was divided, with 50% applied before sowing and the remainder top-dressed at the jointing stage. Field management adhered to local high-yield agronomic practices.

### 2.2. Grain Flour Quality Analysis

Wheat grains were milled into flour using a flour mill with a 70GG mesh sieve (LRMM804; Buhler, Wuxi, China). A near-infrared grain analyzer (DA7200; Perten, Sweden) was used to determine the wheat grain protein content, while a gluten analyzer (MJ-III; Dacheng Optoelectronic Technology Co., Ltd., Huizhou, Guangdong, China) was used to measure the wet gluten content. Dough rheological properties were assessed using the Brabender Farinograph-E with 300 g bowl and Extensograph-E devices (Anton Paar TorqueTec GmbH, Duisburg, Germany). The water absorption rate, development time, and stability time were measured by Farinograph-E, while the extension resistance and energy area were determined by Extensograph-E devices. The solvent retention capacities (SRCs) of water, sucrose, sodium carbonate, and lactic acid were evaluated using SRC-Chopin (Chopin Technologies, Villeneuve-la-Garenne, France). The protein secondary structure was analyzed by Fourier transform infrared spectroscopy (Vertex 70; Bruker Daltonics, Bremen, Germany) following the methods described by Yan et al. [24].

### 2.3. Flour Proteome Analysis

Proteins were extracted from flour samples (each sample comprising three biological replicates) using SDT buffer (4% SDS, 100 mM Tris-HCl, pH 7.6). Protein digestion was performed using a filter-aided sample preparation protocol [25]. Upon completion of digestion, the peptide content was estimated using the ultraviolet light spectral density at 280 nm, using an extinction coefficient of 1.1 for a 0.1% (g/L) solution [25].

Liquid chromatography–tandem mass spectrometry analysis of peptide segments was performed using a timsTOF Pro mass spectrometer (Bruker Daltonics, Billerica, MA, USA) coupled with a NanoElute system (Bruker Daltonics) for 60, 120, and 240 min. The analysis was performed at Applied Protein Technology Co., Ltd., Shanghai, China, following the method described by Feng et al. [23]. In brief, sample separation used a reversed-phase trap column (Acclaim PepMap 100, 100 μm × 2 cm, nanoViper C18; Thermo Fisher Scientific, Waltham, MA, USA), connected to a C18 reversed-phase analytical column (Thermo Fisher Scientific Easy Column, 10 cm long, 75 μm inner diameter, 3 μm resin). Raw mass spectrometry data from samples were analyzed using MaxQuant v.1.5.3.17 for protein identification and quantification. The UniProt database (http://www.uniprot.org/, accessed on 25 November 2022) was used for protein sequence matching, with the false discovery rate for both proteins and peptides set at ≤0.01. Protein quantification incorporated both razor and unique peptides, using the label-free quantification intensity method, with a 2 min time window (match between runs) and the minimum ratio count set at 1. Significantly differentially expressed proteins (DEPs) between treatments were identified with a *p*-value <0.05 and a ratio of either <0.83 or >1.2.

### 2.4. WGCNA

The homologous genomes (A, B, and D genomes) of differential proteins were obtained by querying the UniProt and Ensembl Plants (http://plants.ensembl.org/index.html, accessed on 8 March 2023) databases. The transcript expression measurement of these genes, encompassing various developmental stages and organs, was retrieved from the expVIP database (http://www.wheat-expression.com/, accessed on 11 March 2023). The co-expression network was constructed using the WGCNA package in R v4.2.2 [26,27]. To ensure biological significance, genes with expression values < 1 were filtered out. The WGCNA soft threshold power (β) was set at 6 to analyze the scale-free topology. The topological overlap measure (TOM) was calculated from the adjacency matrix and then converted into a dissimilarity matrix (1-TOM). A cluster dendrogram was created using hierarchical clustering, and the gene module was detected via the dynamic tree cut algorithm with a threshold minimum module size of 30 genes [28]. The co-expression network of key modules was visualized using Cytoscape v3.9.1 [29]. Hub genes were identified using the maximal clique centrality (MCC) algorithm of the CytoHubba plugin in Cytoscape [30]. Motif annotation of promoters (2 kb region upstream of the start codon of hub genes) was performed using the PlantCARE database (http://bioinformatics.psb.ugent.be/webtools/plantcare/html, accessed on 15 September 2023).

### 2.5. Gene Expression Analysis Using Quantitative Real-Time PCR (qRT-PCR)

qRT-PCR was performed using the ChamQ SYBR qPCR Master Mix (Vazyme Biotech Co., Ltd., Nanjing, China) on a QuantStudio 5 Dx Real-Time PCR System (Thermo Fisher Scientific). The 2^−ΔΔCt^ method was used to calculate the relative gene expression levels across samples [31], with β-actin serving as the internal reference gene. The primers used for qRT-PCR are listed in Table S1.

### 2.6. Data Analysis

Data were analyzed and evaluated by Statistical Program for Social Sciences (SPSS 22.0) software, and the results are shown as means ± standard deviation (*n* = 3). The independent-samples *t*-test through the Compare Means function in SPSS was used to distinguish differences between various treatment groups, and *p* < 0.05 was considered to be statistically significant.

## 3. Results

### 3.1. Grain Protein Content and Quality Parameters Under Different Nitrogen Treatments

The wheat cultivar ‘KX 3302’ exhibited elevated total grain protein and wet gluten contents in the HN treatment group, but showed reduced values in the LN treatment group (Table 1). Correspondingly, the dough rheological characteristics, which are important quality characteristics of wheat flour [32], including development time, stability time, energy area, and extension resistance, of the dough from the HN treatment group demonstrated higher values compared with the dough from the LN treatment group. For instance, compared with LN treatment, HN treatment increased the development time and stability time of flour by 155.10% and 30.90%, respectively. Furthermore, regarding protein secondary structure, wheat grown under HN conditions displayed higher values of β-sheets (46.81%) and β-turns (29.46%), whereas wheat cultivated under LN conditions showed a higher irregular curl content (15.34%). The difference in β-sheets, β-turns, and irregular curl between HN and LN treatments reached a significant level at *p* < 0.05.

SRC is used to assess flour functionality related to specific flour components, with sucrose SRC associated with gliadin content, lactic acid SRC linked to glutenin characteristics, and water SRC influenced by all these flour components [33]. In this study, all four types of SRC of flour from the HN treatment exhibited higher values. Ultimately, wheat grown under HN conditions produced bread with a greater volume and scores, while those under LN conditions yielded lower values. These findings suggest that HN application increases the grain protein content, improves the protein structure, enhances the dough strength, and elevates the bread quality.

### 3.2. Grain Protein Components Identified by Proteomic Analysis

**Figure 1:**
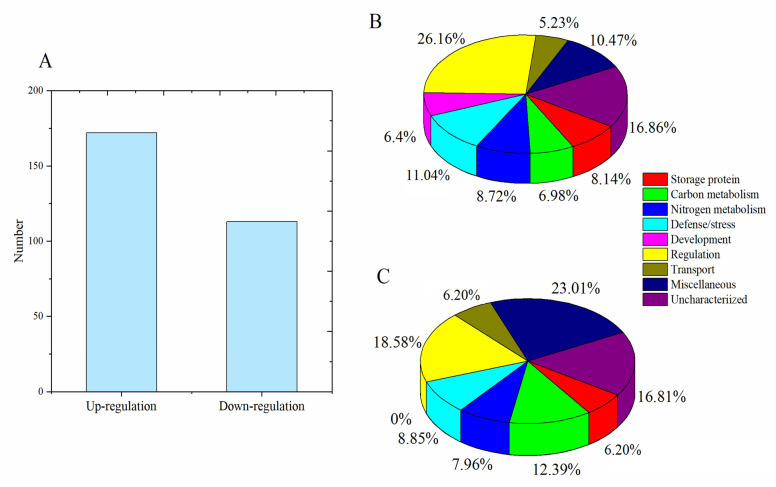
Number of differentially expressed proteins (DEPs) and their classification. (**A**) Number of DEPs between high-nitrogen (HN) and low-nitrogen (LN) treatments. (**B**) Categorization of upregulated DEPs. (**C**) Categorization of downregulated DEPs.

**Figure 2:**
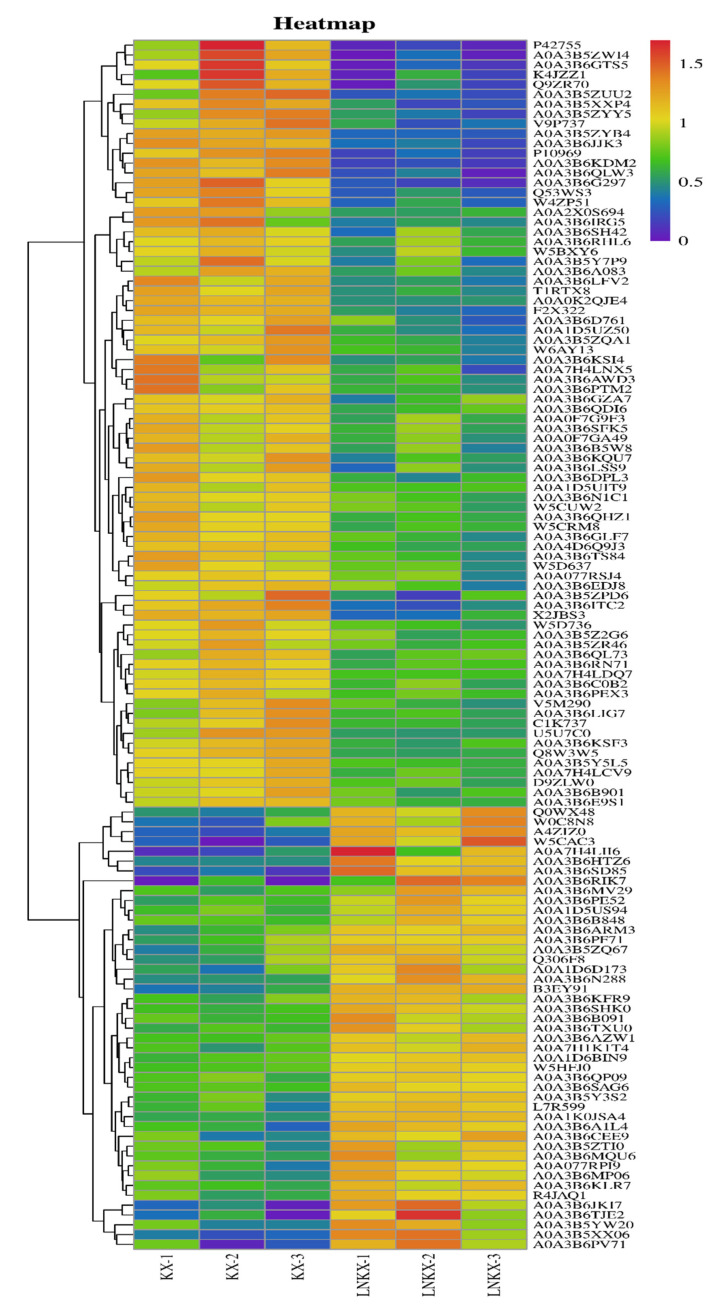
Cluster heatmap of DEPs with fold change ≥1.5. HNKX, ‘Kexing 3302’ wheat under high-nitrogen treatment; LNKX, ‘Kexing 3302’ wheat under low-nitrogen treatment. Red represents relatively high expression abundance, while blue represents relatively low expression abundance.

A total of 285 DEPs were identified in wheat grains under both HN and LN conditions (Table S2), including 172 DEPs upregulated in HN treatment (Figure 1). Among these upregulated proteins in HN treatment, 14 were storage proteins (8.14%) and 12 were involved in carbon metabolism (6.98%), 15 in nitrogen metabolism (8.72%), 19 in defense/stress (11.04%), 11 in development (6.40%), and 45 in regulation (26.16%). Upregulated storage proteins in HN treatment included three HMW-GSs (W6AY13, Q1KL95, and A9YSK4), three LMW-GSs (V9P737, Q8W3W5, and X2JBS3), four α/β-gliadins, and three γ-gliadins. Conversely, the upregulated proteins in LN treatment comprised 7 storage proteins (6.20%), 14 proteins involved in carbon metabolism (12.39%), 9 in nitrogen metabolism (7.96%), 10 in development (8.85%), and 21 in regulation (18.58%). Storage proteins upregulated in the LN group comprised three LMW-GS, two α-gliadins, and two γ-gliadins. In addition to storage proteins, numerous metabolic proteins were upregulated under HN treatment, suggesting that nitrogen fertilizer application increased the content of many metabolic proteins and stress/defense proteins in grains. For instance, the expression intensities of 1,4-alpha-glucan branching enzyme (A0A3B6RST4) and Em protein (W4ZP51) were 5.04 × 10^6^ and 1.44 × 10^7^ under HN treatment, respectively, compared with 3.65 × 10^6^ and 4.64 × 10^6^ under LN treatment. The expression levels of two peroxidases A0A3B6DPL3 and A0A077S7C3 were 1.99- and 1.25-fold higher under HN treatment than under LN treatment. Additionally, the results revealed that 29 (16.86%) DEPs of the upregulated proteins under HN treatment were uncharacterized, suggesting their potential involvement in mediating nitrogen-efficient promotion of grain protein accumulation. For instance, protein A0A3B6KKC0 exhibited substantial abundance (2.07 × 10^6^~2.97 × 10^6^ intensity), while A0A3B5ZWI4 demonstrated an 8.83-fold increase in expression under HN conditions compared to low-nitrogen (LN) treatment. DEPs with a fold change ≥1.5 were clustered in the heatmap (Figure 2), with 78 DEPs upregulated in HN treatment and 45 DEPs upregulated in LN treatment. It is clear that the protein cluster including P42755, AOA3B5ZW14, A0A3B6GTS5, K4JZZ1, Q9ZR70, A0A3B5ZUU2, and A0A3B5XXP4 has a relatively low expression abundance in LN treatment, while the protein cluster containing Q0WX48, W0C8N8, A4ZIZ0, and W5CAC3 has a relatively low expression abundance in HN treatment.

### 3.3. WGCNA and Screening of Hub Genes

A total of 348 genes encoding DEPs (with fold change ≥1.5) were identified using the UniProt and Ensembl Plants databases (Table S3). The transcript expression measurements were obtained by querying the expVIP database (Table S4). Following gene expression data filtering and screening, a total of 293 genes were used in network construction with a minimal module size of 30 genes and merge cut height of 0.20 (Figure S1). Three gene modules were identified: turquoise (130 genes), blue (123 genes), and gray (40 genes). Then, the module eigengenes of different modules were calculated, followed by a correlation analysis between module eigengenes and samples where *p* < 0.05 was considered statistically significant, and defined as hub modules [34]. A heatmap was generated to visualize the strength of the correlations between module eigengenes and samples (Figure S2). The blue module exhibited a strong correlation with grain samples, while the turquoise module showed a good correlation with root and seed samples (Figure S2). Consequently, we hypothesized that the blue module is associated with grain protein expression, and thus the blue module was the focus of subsequent analysis.

Genes in the blue module revealed enrichment in the three Gene Ontology (GO) categories of “biological process”, “molecular function”, and “cellular component” (Figure S3). In the “biological process” category, seventeen genes were enriched in response to abscisic acid (ABA) (GO:0009737), eight genes in protein folding (GO:0006457), six genes in response to hydrogen peroxide (GO:0042542), and three genes in protein poly-ADP-ribosylation (GO:0070212). For the “cellular component” category, five genes were primarily enriched in the extracellular region (GO:0005576), three genes in the integral component of the chloroplast outer membrane (GO:0031359), and three genes in the plasmodesma (GO:0009506). Regarding the “molecular function” category, the most abundant terms were unfolded protein binding (GO:0051082), with eight genes significantly enriched in nutrient reservoir activity (GO:0045735), six genes in protein self-association (GO:0043621), and two genes each in protein phosphatase binding (GO:0019903) and glutathione disulfide oxidoreductase activity (GO:0015038).

**Figure 3:**
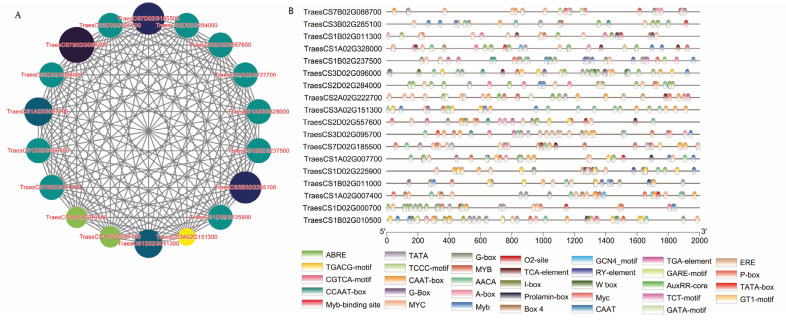
Gene interaction network. (**A**) Network diagram of the core genes selected from the blue module. (**B**) Promoter *cis*-element analysis of the core genes. Larger circles and darker colors indicate higher rankings of genes within the core genes of the module.

Subsequently, the genes in the blue module were input into Cytoscape for gene interaction network visualization. The MCC algorithm of the CytoHubba plugin was used to identify core genes, with the top 18 selected as core genes for the blue module (Figure 3). In the core gene network diagram, larger circles and darker colors indicate higher rankings among the module’s core genes. Homologous genes in *Arabidopsis thaliana* were functionally annotated using the Ensembl Plants and the Arabidopsis Information Resource databases (Table 2). For example, TraesCS7B02G088700, a high-ranking gene in the core gene network, corresponds to the *Arabidopsis* ortholog AT1G74720, encoding a transmembrane protein with four C2 domains. AT4G10250, the ortholog of TraesCS7D02G185500, is a small heat-shock protein localized to the inner membrane. TraesCS3B02G285100 shares homology with AT3G02480 in *Arabidopsis*, functioning as a regulatory component in the downstream pathway of light-induced leaf senescence. Furthermore, the *Arabidopsis* ortholog AT1G77140 of TraesCS1B02G011300 represents a peripheral membrane protein associated with microsomal membranes and involved in protein trafficking to the vacuole. These findings suggest that multiple metabolic pathways are involved in grain protein accumulation in response to nitrogen fertilizer application.

To investigate the response of these genes to nitrogen fertilizer, an analysis of promoter *cis*-acting elements was conducted on 18 core genes. As illustrated in Figure 3, 17 genes possessed elements involved in the ABA response (ABA response element [ABRE] motif) and light response (G-box); all of the genes (except for TraesCD1B02G011300) contained TATA or AACA motifs, and additional motifs, such as O2 site, GCN4_motif, and RT-element. For instance, six AACA motifs, five ABREs, three G-boxes, and six CAAT-boxes were identified in TraesCS3A02G151300. In TraesCS1B02G011300, *cis*-elements involved in endosperm expression (GCN4_motif), regulation of zein metabolism (O2 site), and ABRE were observed. These findings suggest the possibility of similar regulatory factors governing the expression of these genes, thereby promoting the accumulation of their corresponding proteins.

### 3.4. Gene Expression Verification

**Figure 4:**
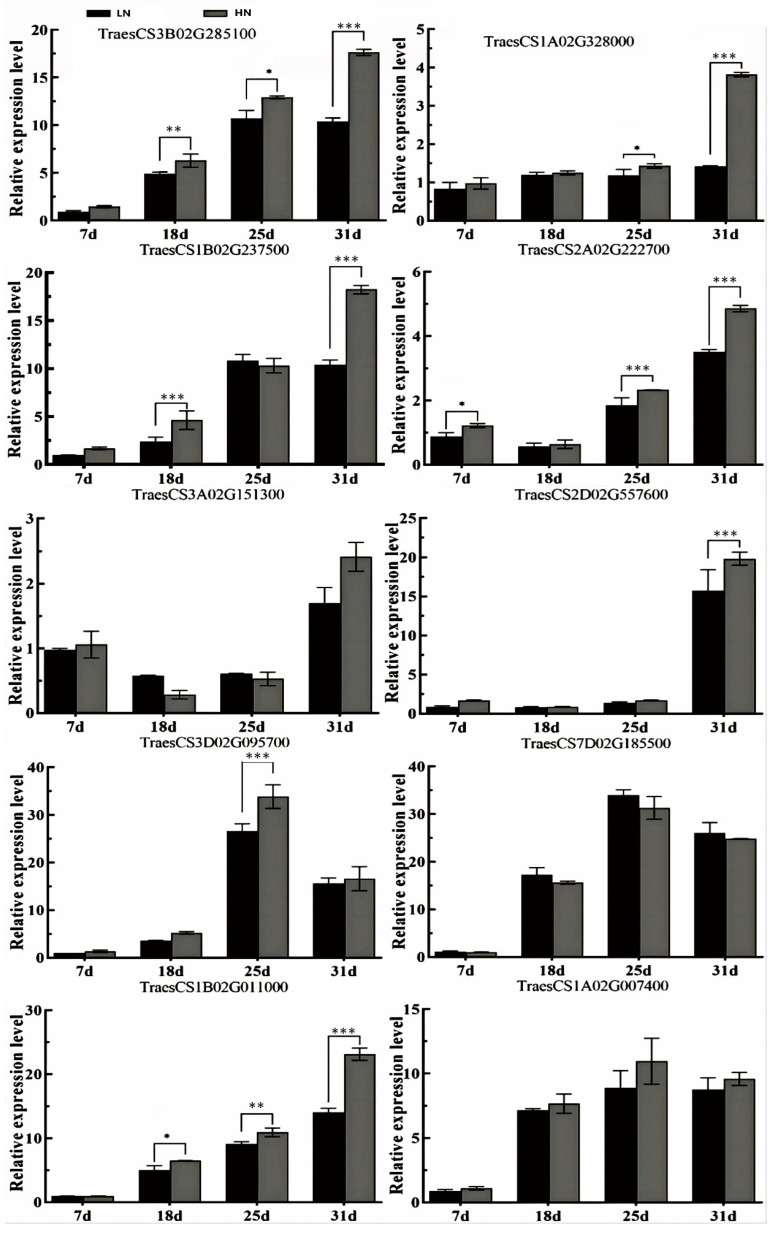
Relative expression levels of core genes in wheat grain under different nitrogen treatments. Vertical bars above the columns indicate the standard deviation of the mean. *, **, and *** above the columns indicate significant differences at *p* < 0.05, *p* < 0.01, and *p* < 0.001 level, respectively.

To validate the role of the identified core genes in promoting protein accumulation in response to nitrogen fertilizer application, qRT-PCR was used to analyze the expression patterns of these genes during grain filling under varying nitrogen fertilizer levels. These genes exhibited similar expression profiles during grain filling, with the majority gradually increasing and reaching their peak expression levels in the late stage of grain filling (Figure 4). Furthermore, the majority of genes exhibited elevated expression levels under HN treatment compared with LN treatment; for instance, at 18, 25, and 31 days after anthesis (DAA), TraesCS3B02G85100 expression levels were 1.28, 1.21, and 1.70 times higher under HN treatment. Similarly, the average expression levels of TraesCS7D02G185500 and TraesCS3A02G151300 at 18 and 25 DAA were 1.10 and 1.59 times higher under HN treatment than under LN treatment. This relatively high expression level under HN treatment may contribute to increased grain protein accumulation.

To elucidate the regulatory mechanisms of wheat grain proteins’ efficient response to nitrogen fertilizer, the transcription factors of three genes (TraesCS1B02G237500, TraesCS1B02G011000, and TraesCS1A02G007400) were predicted by PlantRegMap (https://plantregmap.gao-lab.org/index-chinese.php, accessed on 25 March 2023). The predicted transcription factors for the TraesCS1B02G237500 gene were Traes_4BL_79192DE.1 and Traes_1BL_A5A91DE10.1, belonging to the transcription factor DOF and Trihelix protein families, respectively (Table 3). For the TraesCS1B02G011000 gene, the predicted transcription factors were TRAES3BF0974000050CFD_t1 and Traes_2AS_40FA27AE7, belonging to the ARF and MYB protein families, respectively. In addition, for the TraesCS1A02G007400 gene, the transcription factors were Traes_4BL_32D8155C6 and Traes_4BS_373BDBA94, belonging to the transcription factor G2-like and NAC protein families, respectively.

**Figure 5:**
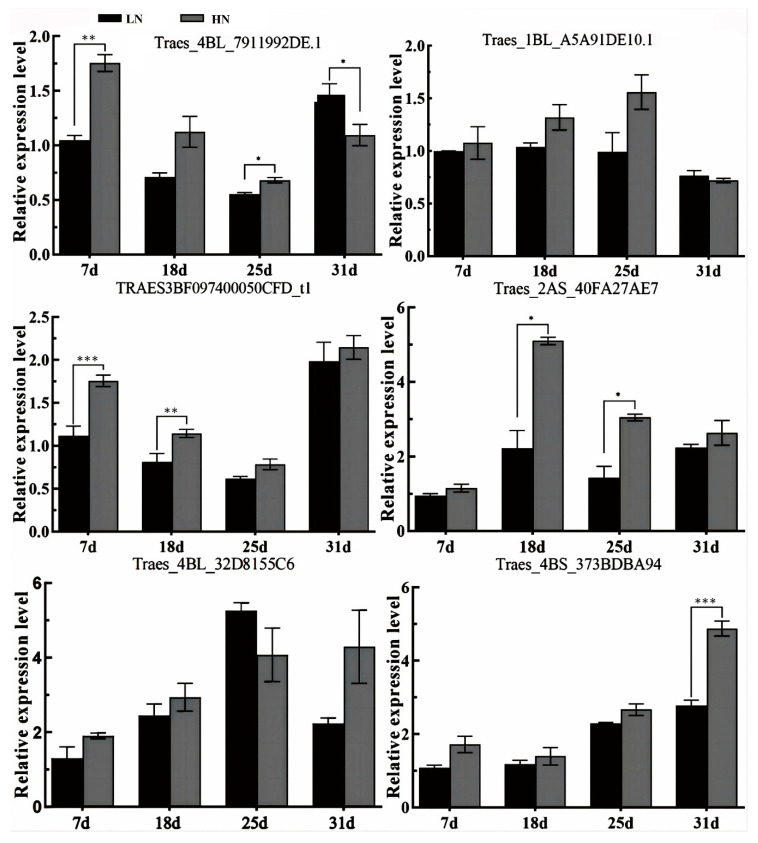
Relative expression levels of predicted transcription factors of core genes. Vertical bars above the columns indicate the standard deviation of the mean. *, **, and *** above the columns indicate significant differences at *p* < 0.05, *p* < 0.01, and *p* < 0.001 level, respectively.

At 7, 18, and 25 DAA, the expression levels of Traes_4BL_79192DE.1 and Traes_1BL_A5A91DE10.1 demonstrated higher values under HN treatment than under LN treatment (Figure 5). Regarding the two transcription factors of TraesCS1B02G011000, TRAES3BF0974000050CFD_t1 exhibited lower expression levels at 18 and 25 DAA, while Traes_2AS_40FA27AE7 reached its peak at 18 DAA during grain filling. Nevertheless, both transcription factors displayed higher expression values under HN treatment relative to LN treatment. In addition, the transcription factors Traes_4BL_32D8155C6 and Traes_4BS_373BDBA94 of the TraesCS1A02G007400 gene demonstrated an increasing trend throughout grain filling. Moreover, the expression levels of these two transcription factors under HN treatment surpassed those under LN treatment. The comparable expression patterns of transcription factors and their target genes under varying nitrogen levels suggest that these transcription factors positively regulate gene expression.

## 4. Discussion

The nitrogen fertilizer application rate significantly influences the wheat grain protein content and flour-processing quality. Higher nitrogen application rates have been associated with an increased wheat grain protein content, dough stability time, and bread-making quality [22,35,36]. In this study, HN treatment significantly enhanced the grain protein content, wet gluten content, stability time, and bread-making quality. The secondary protein structure is crucial in determining gluten network characteristics [37,38]. Our findings revealed that the β-sheet structure (44.55–46.18%) constituted the highest percentage among the structural components, consistent with previous research [39]. β-sheets and β-turns were higher under HN treatment, while irregular curls were higher under LN treatment. Thus, the increased β-sheet content under HN treatment may contribute to improved gluten network characteristics and an enhanced bread quality. Improvements in the stability and cohesiveness of the gluten network correlate with an increase in β-sheet structure and larger polymeric proteins [40]. The primary structure of proteins serves as the fundamental determinant of their higher-order structural conformations. The observed increase in β-sheet content under HN conditions may be attributed to the accumulation of β-sheet-enriched protein components, particularly HMW-GS. Previous experimental evidence demonstrated that genetic silencing of HMW-GS resulted in a significant reduction in β-sheet content and an increase in irregular coil structures compared to wild-type controls [41].

Nitrogen fertilizer application affects not only the protein content but also its composition [12,13]. Compared with conventional cultivation patterns, reduced nitrogen rates significantly downregulated HMW-GSs (11), LMW-GSs (8), α/β-gliadins (5), and γ-gliadins (2) [14]. We observed a higher abundance of HMW-GSs (3) and LMW-GSs (3) under HN treatment, while some LMW-GSs (3) and γ-gliadins (2) showed a higher abundance under LN treatment. These findings suggest that different protein components respond variably to nitrogen levels and contribute differently to gluten network formation. HMW-GSs, LMW-GSs, and α/β-gliadins, which were highly expressed under HN conditions, collectively promote the formation of high-quality gluten networks, which was evidenced by improved bread scores compared with the LN treatment. Furthermore, 172 DEPs were identified, including not only storage proteins but also those involved in carbon/nitrogen metabolism and regulatory functions. This indicates that apart from storage proteins, certain metabolic proteins participate in gluten network formation. Wang et al. [5] reported that stress-associated and metabolic proteins interacted with the wheat dough matrix. In our study, the serine protease inhibitor (A0A3B6KQL2) and peroxidase (A0A3B6DPL3) were highly expressed under HN conditions, suggesting that these enzyme proteins contribute to the formation of strong gluten network structures. Research has shown that dough treated with peroxidase exhibited increased hardness and decreased adhesiveness [42]. Thus, in addition to storage proteins, future research should explore the synthesis of metabolic proteins that respond efficiently to nitrogen and their effects on food quality.

The application of nitrogen fertilizer promotes the accumulation of grain storage proteins via the increased expression of related genes. Zhang et al. [22] demonstrated that HN levels significantly enhanced glutamine synthetase gene expression and activity. Under nitrogen-deficient conditions, reduced expression of genes associated with amino acid synthesis and protein transport led to a 17–42% decrease in ornithine, cysteine, and aspartate in metabolic components, resulting in an 18.6% reduction in grain protein content [43]. WGCNA is an effective method for identifying genes related to target traits [44,45]. In this study, a blue module comprising 123 genes was identified as closely related to grain protein. GO analysis indicated that the biological processes of these genes primarily involve the ABA response, protein folding, and hydrogen peroxide response. Analysis of *cis*-acting elements in the promoter region of core genes revealed that most genes share *cis*-acting elements, such as those involved in the ABA response (ABRE), light response (G-box), and zein metabolism regulation (O2 site). These findings suggest that nitrogen fertilizer influences grain protein accumulation through multiple biological processes, with the ABA response potentially playing a crucial role. ABA can regulate the glutenin fraction content and glutenin macropolymer size distribution, thereby improving the nutrient quality of wheat [46]. In addition, several motifs related to storage protein regulation and endosperm expression have been identified, including AACA, TATA, and GCN4_motif [47,48,49]. Gene expression verification confirmed that the identified genes exhibited similar expression patterns during grain filling under different nitrogen fertilizer levels. Furthermore, the comparable expression patterns of transcription factors Traes_4BL_32D8155C6 and Traes_4BS_373BDBA94 and their target gene TraesCS1A02G007400 under varying nitrogen levels suggest that these transcription factors positively regulate gene expression. Further research is necessary to elucidate the regulatory mechanisms by which transcription factors promote the efficient response of grain protein to nitrogen fertilizer. It must be emphasized that while the current approach establishes a more direct and robust connection between transcriptional profiles and grain protein accumulation, some critical regulatory factors playing a pivotal role in regulating grain protein deposition during the seed development process may potentially be overlooked.

## 5. Conclusions

The grain protein content, wet gluten content, stability time, and energy area exhibited higher values under the HN treatment. Concurrently, the β-sheet content of the secondary protein structure increased, while the irregular curl content decreased. Grain proteomic analysis revealed that both storage proteins and certain metabolic proteins contribute to dough network formation. WGCNA identified 18 core genes, with most containing ABREs, storage protein regulation elements, and endosperm expression elements in their promoter regions. qRT-PCR analysis confirmed similar expression patterns for the majority of these genes during grain filling under varying nitrogen fertilizer levels. By starting from mature grain proteome profiling and progressing to WGCNA of corresponding coding genes of differential protein components, this study establishes a more robust relationship between gene expression and protein composition and provides valuable insights into the enhancement of grain protein accumulation and dough quality by nitrogen fertilizer application.

## Figures and Tables

**Table 1:** Grain protein content and quality parameters under different nitrogen treatments.

Traits		HN	LN
Protein and gluten content	Protein content (%)	14.58 ± 0.47 ^a^	13.01 ± 0.28 ^b^
	Wet gluten content (%)	31.00 ± 1.56 ^a^	27.18 ± 1.04 ^b^
	Gluten index	0.97 ± 0.01 ^a^	0.95 ± 0.03 ^a^
Rheological properties	Water absorption rate (%)	68.00 ± 1.84 ^a^	66.60 ± 1.31 ^a^
	Development time (min)	25.00 ± 0.01 ^a^	9.80 ± 5.54 ^b^
	Stability time (min)	23.00 ± 0.17 ^a^	17.57 ± 1.44 ^b^
	Energy area (cm^2^)	183.00 ± 17.00 ^a^	176.7 ± 31.66 ^a^
	Extension resistance (EU)	629.00 ± 3.00 ^a^	562.7 ± 27.15 ^b^
	Extensibility (mm)	155.50 ± 1.50 ^a^	162.33 ± 7.76 ^a^
Protein secondary structure	β-sheets (%)	46. 81 ± 0.004 ^a^	44.55 ± 0.013 ^b^
	α-helix (%)	12.29 ± 0.007 ^a^	12.82 ± 0.002 ^a^
	β-turns (%)	29.46 ± 0.009 ^a^	27.29 ± 0.011 ^b^
	Irregular curl (%)	11.44 ± 0.011 ^b^	15.34 ± 0.005 ^a^
Solvent retention capacity	Water (%)	93.54 ± 0.72 ^a^	89.98 ± 3.01 ^a^
	Sucrose (%)	138.54 ± 3.17 ^a^	132.84 ± 1.27 ^b^
	Sodium carbonate (%)	120.03 ± 0.13 ^a^	109.11 ± 1.68 ^b^
	Lactic acid (%)	122.48 ± 0.94 ^a^	116.50 ± 6.45 ^a^
Bread-making quality	Volume (cm^3^)	707.50 ± 6.36 ^a^	690.00 ± 1.41 ^a^
	Score	76.90 ± 0.26 ^a^	71.20 ± 0.06 ^b^

Notes: Data represent the mean ± standard deviation. Within the same row, mean values followed by different lowercase letters indicate significant differences at *p* < 0.05 level.

**Table 2:** Functional annotation of homologous genes in *Arabidopsis thaliana* corresponding to core genes in the blue module.

Core Gene ID	Homologous Gene ID in *A. thaliana*	Gene Function
TraesCS7B02G088700	AT1G74720	Encodes a putative transmembrane protein carrying four C(2) domains; involved in organ development.
TraesCS3B02G285100	AT3G02480	LEA protein that is upregulated by ABA, NaCl, and light deprivation.
TraesCS1B02G011300	AT1G77140	A peripheral membrane protein that associates with microsomal membranes, likely to function in the transport of proteins to the vacuole.
TraesCS1A02G328000	AT5G22470	PARP3 is one of three canonical PARPs in Arabidopsis.
TraesCS1B02G237500	AT3G51810	Encodes an ABA-inducible protein that accumulates during seed maturation.
TraesCS3D02G096000	AT1G66180	Encodes a putative aspartyl protease.
TraesCS2D02G284000	AT5G50590	Encodes a putative hydroxysteroid dehydrogenase
TraesCS2A02G222700	AT2G34740	protein phosphatase 2C family protein.
TraesCS3A02G151300	AT1G64110	Target promoter of the male germline-specific transcription factor DUO1.
TraesCS2D02G557600	AT2G26530	Pheromone receptor-like protein involved in the early elicitor signaling events.
TraesCS3D02G095700	AT1G74490	Protein kinase superfamily protein.
TraesCS7D02G185500	AT4G10250	Columbia endomembrane-localized small heat shock protein.
TraesCS1A02G007700	AT4G31580	Encodes a Serine/arginine-rich (SR) protein RSZp22.
TraesCS1D02G225900	AT1G11960	Calcium channel that is phosphorylated by BIK1 in the presence of PAMPS and required for stomatal immunity.
TraesCS1B02G011000	AT1G07560	Leucine-rich repeat protein kinase family protein.
TraesCS1A02G007400	AT2G01150	Encodes a RING-H2 finger protein.
TraesCS1D02G000700	AT2G32160	Methyltransferase gene.
TraesCS1B02G010500	AT2G01150	Encodes a RING-H2 finger protein.

**Table 3:** Predicted transcription factors of core genes.

Gene ID	Transcription Factor ID	Transcription Factor Family
TraesCS1B02G237500	Traes_4BL_7911992DE.1	Dof
	Traes_1BL_A5A91DE10.1	Trihelix
TraesCS1B02G011000	TRAES3BF097400050CFD_t1	ARF
	Traes_2AS_40FA27AE7	MYB
TraesCS1A02G007400	Traes_4BL_32D8155C6	G2-like
	Traes_4BS_373BDBA94	NAC

## Data Availability

The original contributions presented in the study are included in the article/Supplementary Materials, further inquiries can be directed to the corresponding authors.

## Supplementary Materials

The following supporting information can be downloaded at https://www.mdpi.com/article/10.3390/foods14091481/s1, Table S1. List of primers used in this study. Table S2. Differentially expressed proteins in grains between high- and low-nitrogen treatments. Table S3. Genes encoding differentially expressed proteins. Table S4. Expression levels of related genes downloaded from public databases. Figure S1. Gene clustering and module segmentation. Figure S2. Correlation heatmaps of gene co-expression network modules and different samples. Figure S3. Gene Ontology enrichment analysis of blue module genes.
