# Supplementary figures and images for: Comparative Proteome and Weighted Gene Co-Expression Network Analyses Uncover the Mechanism of Wheat Grain Protein Accumulation in Response to Nitrogen Fertilizer Application
Source: Foods. 2025 Apr 24;14(9):1481. doi: 10.3390/foods14091481 (PMC12071265; doi:10.3390/foods14091481)

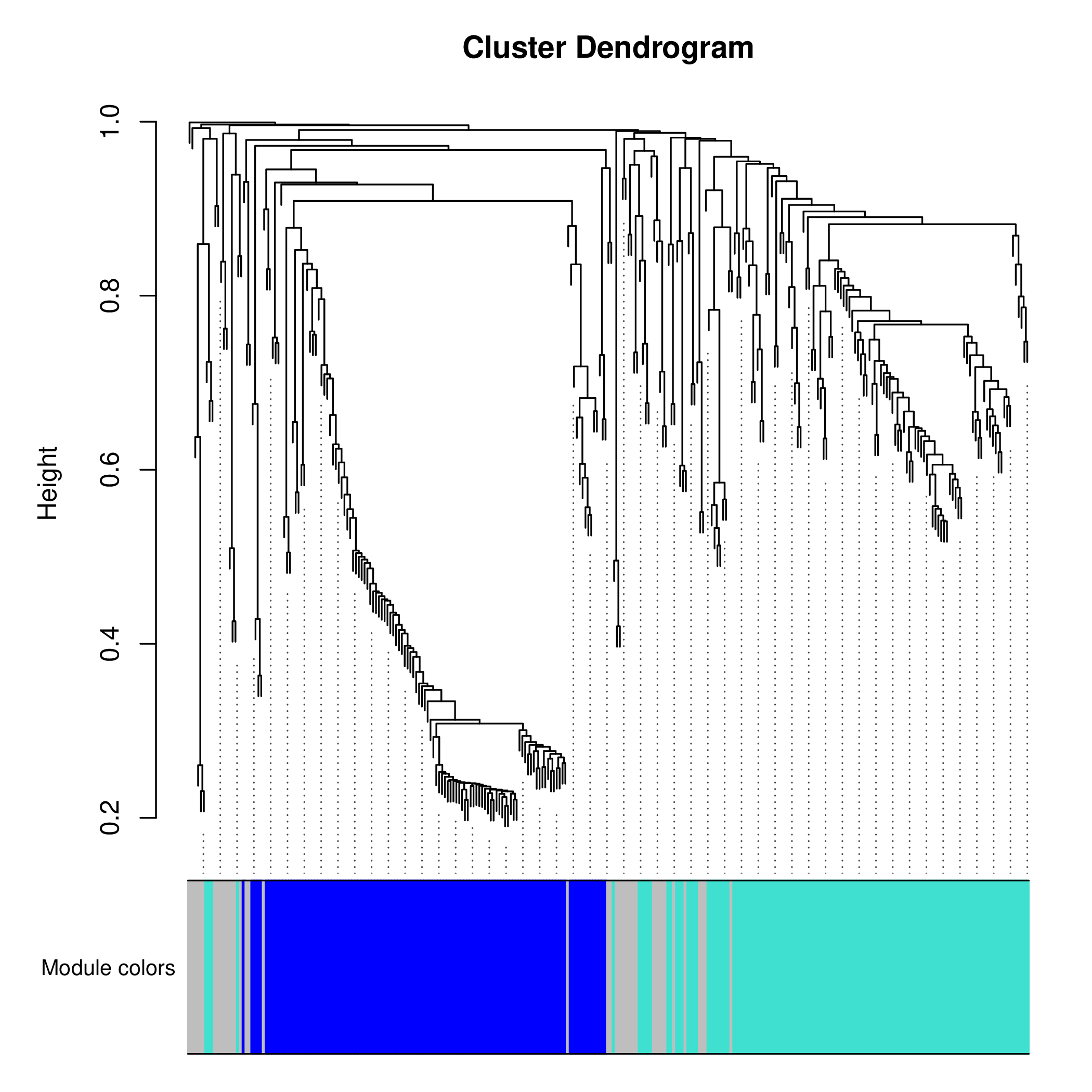

Supplement: Supplementary file 1 [file foods-14-01481-s001.zip › Fig S1.png]

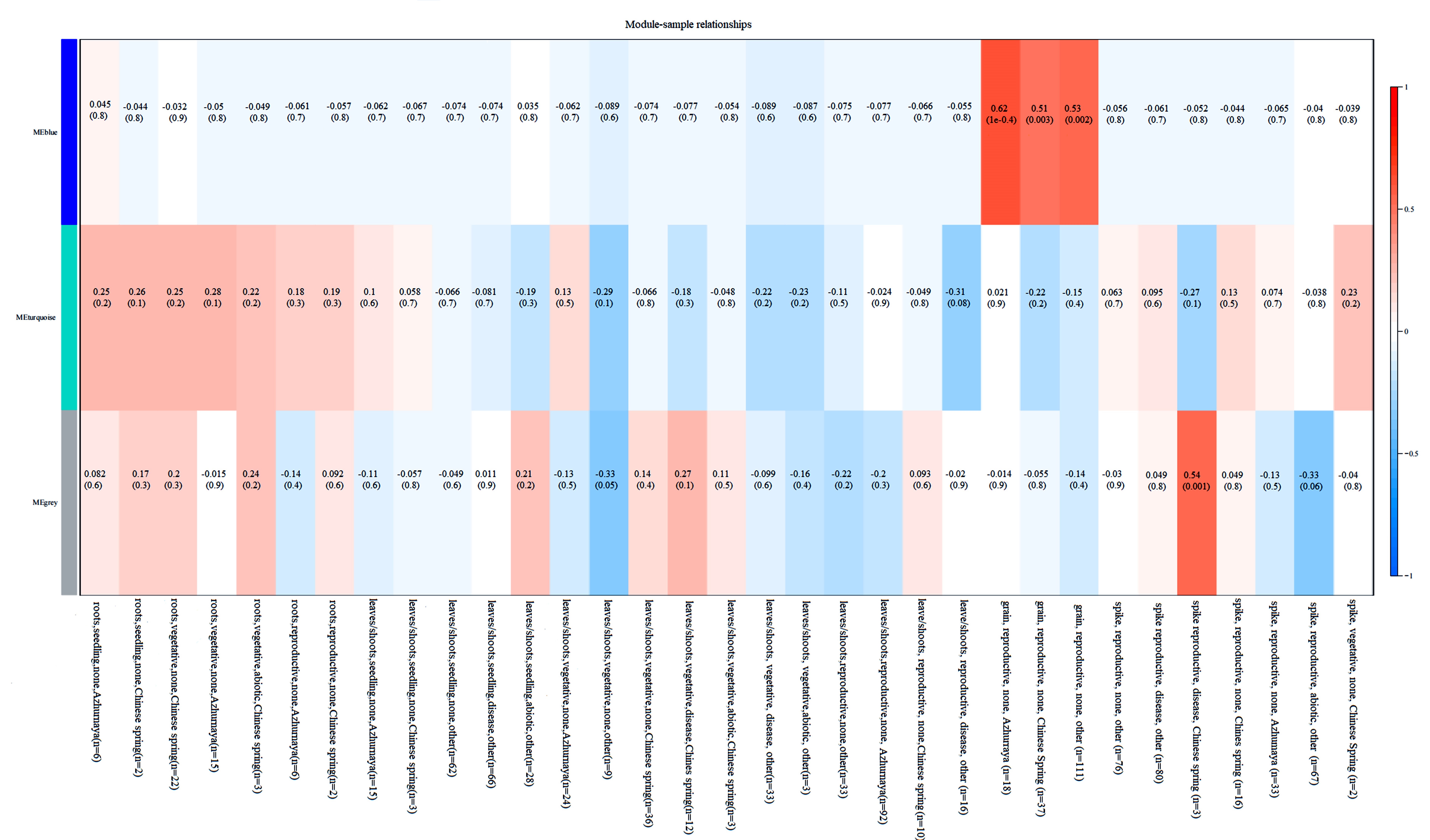

Supplement: Supplementary file 1 [file foods-14-01481-s001.zip › Fig S2.jpg]

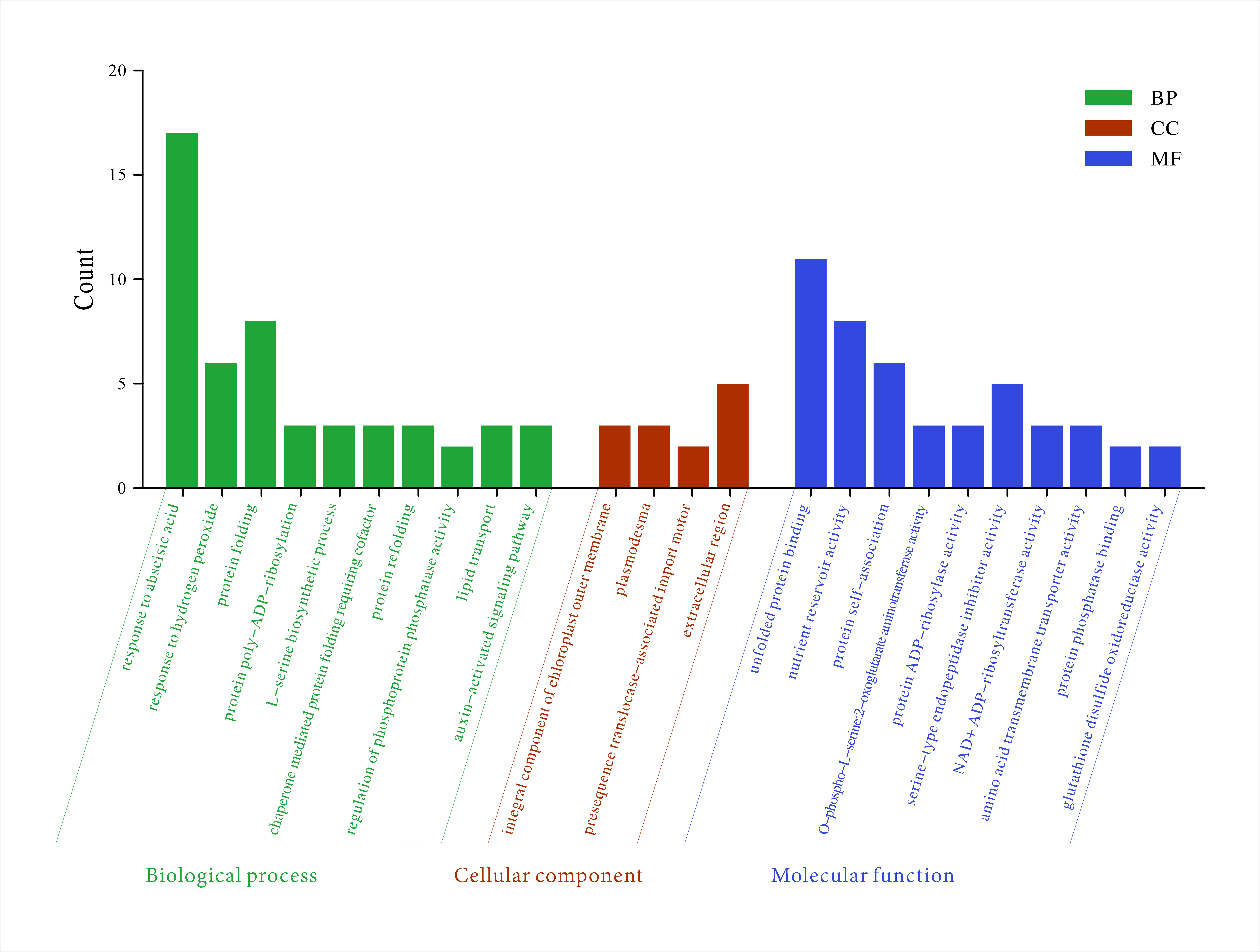

Supplement: Supplementary file 1 [file foods-14-01481-s001.zip › Fig S3.jpg]
